# A Machine-Learning-Based Method for Identifying the Failure Risk State of Fissured Sandstone under Water–Rock Interaction

**DOI:** 10.3390/s24175752

**Published:** 2024-09-04

**Authors:** Jinbo Qu, Cheng Song, Jinwen Bai, Guorui Feng, Xudong Shi, Junbiao Ma

**Affiliations:** 1 College of Mining Engineering, Taiyuan University of Technology, Taiyuan 030024, China; 15343480427@163.com (J.Q.); fguorui1216@163.com (G.F.); 15235127229@163.com (X.S.); majbwangyl@163.com (J.M.); 2 Key Laboratory of Shanxi Province for Mine Rock Strata Control and Disaster Prevention, Taiyuan University of Technology, Taiyuan 030024, China; 3 Department of Energy Engineering, Shanxi Institute of Science and Technology, Jincheng 048011, China

**Keywords:** acoustic emission (AE), fissured sandstone, water–rock interaction, precursor information, instability identification, machine learning

## Abstract

The mechanical properties of fissured sandstone will deteriorate under water–rock interaction. It is crucial to extract the precursor information of fissured sandstone instability under water–rock interaction. The potential of each acoustic emission (AE) parameter as a precursor for instability in the failure process of fissured sandstone was investigated in this study. An experimental dataset comprising 586 acoustic emission experiments was established, and subsequent classification training and testing were conducted using three machine learning (ML) models: AdaBoost, MLP, and Random Forest (RF). The primary parameters for identifying the instability risk state of fissured sandstone include acoustic emission ringing count, energy (mV·ms), centroid frequency, peak frequency, Rise Angle (RA), Average Frequency (AF), *b* value, and the natural/saturated state of fissured sandstone: state. To enhance data utilization, a 10-fold cross-validation method was employed during the model training process. The machine learning models were developed and designed to identify the instability risk of fissured sandstone under the natural and saturated states. The results demonstrated that the established RF model was capable of identifying fissured sandstone instability risks with an *accuracy* of 97.87%. Feature importance analysis revealed that state and *b* value exerted the most significant influence on identification results. The Spearman correlation coefficient was utilized to assess the correlation between input features. This study can provide technical support to identify the risk of instability of fissured sandstones under both natural and saturated water conditions. Based on the models developed in this study, it is possible to implement an early warning method for instability in fissured sandstone that meets realistic working conditions. Compared with the traditional empirical and formulaic methods, the machine learning method can more quickly process huge amounts of AE data and accurately identify the damage state of fissured sandstone.

## 1. Introduction

Fissured rock mass, which undergoes water–rock interaction, is widely distributed throughout underground mining. The strength of the rock can be compromised as a result of this interaction [1,2]. The identification of instability precursor information in fissured sandstone and the establishment of an early warning model are crucial for implementing proactive measures to prevent and control instability risks. Acoustic emission (AE) monitoring, being a crucial non-destructive method for monitoring purposes, has been extensively employed in the surveillance of rock deterioration in underground mining, tunneling, and geological hazards [3,4,5]. The prediction of rock mass instability can be facilitated by utilizing specific AE parameters, which can be regarded as precursor information, according to findings from scholarly research. For example, Zhang et al. [6] concluded that the appearance of stress drop, a sudden increase in dissipated strain energy, and a notable decline in the *b* value can be considered precursor information for sandstone instability. According to Niu et al. [7], the *b* value of AE can be considered precursor information for large-scale occurrences throughout a rock body fracture process. Chmel A et al. [8,9] verified the reliability of the *b* value as a recognition of precursor information. Li et al. [10] extracted the fractal dimension of the AE signals during the loading process and found that the changing pattern of the fractal dimension of the AE signals at different loading stages can be used to predict coal–rock dynamics disasters. Zhang et al. [11] concluded that the autocorrelation coefficients of AE counts, energy, and RA showed a significant and pronounced increase before rock rupture. Wang et al. [12] obtained the characteristics of granite crack extension and damage evolution stages by comprehensive analysis of AE signals like ringing count and energy. Yang et al. [5] characterized the development of rock fractures by information on multiple acoustic emission parameters such as amplitude, event number, and energy. The studies above have demonstrated the feasibility of identifying precursors to fissured sandstone instability by analyzing acoustic emission parameters in water–rock interactions.

In recent years, more and more rock mechanics and geology studies have used machine learning as a research method due to its significant advantages in data processing and dealing with nonlinear problems. For instance, Liang et al. [13] used three machine algorithms to predict the stability of a rock column. Wang et al. [14] used multiple machine learning methods to forecast landslide stability based on 77 real-life cases. Su et al. [15] utilized the clustering unsupervised learning method to automatically extract the clustering features of acoustic emission signals, and the outcomes demonstrated that a particular class of AE parameters can serve as the predictor features for hard rock instability. Duan et al. [16] used four machine learning algorithms to develop improved AE quiet period identification methods. Zhou et al. [17] proposed a machine learning algorithm using a particle swarm algorithm combined with a gradient-boosting decision tree to predict the stability of an underground excavated foundation pit. Dong et al. [18] established a convolutional-neural-network-based image recognition model for micro-seismic events and explosion waveforms. Many other scholars used machine learning methods in the identification of damage states and structural health checks of concrete and composites as well [19,20,21,22,23,24,25,26,27]. In summary, some scholars have used physical parameters, AE parameters, or image data as datasets, combined with the use of machine learning for clustering, classification, and regression to achieve rock damage prediction. However, the application of machine learning in predicting rock damage and instability is still in its nascent stage.

The research findings above have significantly contributed to identifying and forecasting precursor information related to coal–rock instability. However, most of these studies primarily focus on specific parameters or a limited number of parameters. It may not comprehensively respond to the acoustic emission information released during rock instability. Furthermore, there is a lack of integration between machine learning methods and research on identifying fissured sandstone instability under water–rock interaction. Therefore, this study focused on fissured sandstones under natural and water-saturated states to investigate their damage response characteristics and identify instability precursor information. Firstly, the original and statistical AE parameters, including ringing count, centroid frequency, peak frequency, RA, AF, energy, and *b* value, were optimized. Subsequently, the evolution characteristics of these parameters with the gradual failure of fissured sandstone were analyzed for use as precursors for identifying instability in fissured sandstone. To avoid any adverse effects caused by different states of the rock body on model performance, both natural and saturated states were adopted as references for analyzing the instability of fissured sandstone. A complete dataset comprising AE parameters for uniaxial compression testing was established to develop three machine learning models that can identify instability risk in fissured sandstone based on this dataset. The performance of each machine learning model was evaluated using multiple metrics to validate the accuracy and reliability of the developed instability risk identification models for fissured sandstone.

## 2. Methodology

### 2.1. Material Preparation and Experiment Setup

#### 2.1.1. Material Preparation

**Figure 1:**
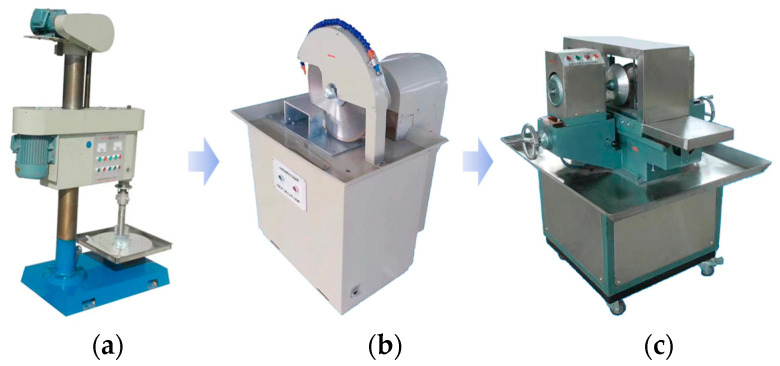
Intact specimen preparation process. (**a**) Drilling; (**b**) cutting; (**c**) polishing.

**Figure 2:**
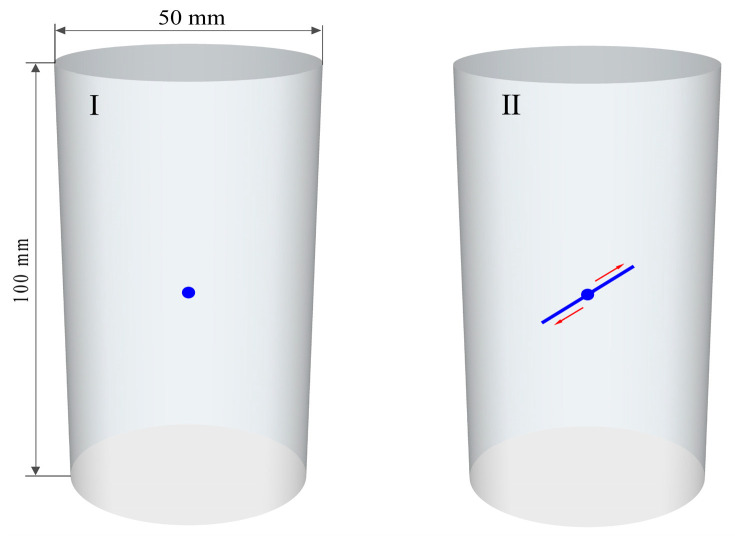
Prefabricated fissure preparation of sandstone specimen. I represents the first step of specimen preparation and II represents the second step. Dot indicates borehole and arrow indicates fissure.

The bulk sandstone samples collected in the field were transported to the laboratory for preparation. The sandstone specimens were prepared following the guidelines provided by the International Society for Rock Mechanics (ISRM) [28]. The specimens were then processed into standard cylindrical forms, with a diameter of 50 mm and a height of 100 mm. The specific preparation process is shown in Figure 1. Subsequently, based on the intact sandstone obtained from the above preparation, sandstone specimens with different prefabricated fracture geometry parameters were further prepared by a mechanical cutting method. A 3 mm diameter diamond drill was used to drill a hole in the center of the intact specimen at the position shown in state I in Figure 2. A circular hole of 3 mm front-to-back through type was formed along the diameter direction of the specimen. Then, a diamond corded saw was passed through the small hole and cut back and forth along the pre-designed direction of fissure distribution until a fissured sandstone specimen was completely produced as shown in state II in Figure 2. It has been experimentally verified that the water-saturated specimens require immersion of the natural specimens in water for 45 days to be obtained. Finally, single-fissured sandstone specimens were obtained for uniaxial compression testing under natural and water-saturated conditions. The length of the fissure was 10 mm, the width was 1.5 mm, and the angle was 45°.

#### 2.1.2. Uniaxial Compression Test of Fissured Sandstone

**Figure 3:**
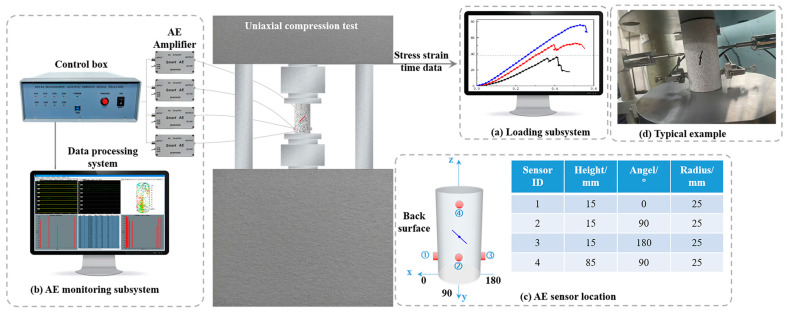
The test system’s schematic diagram layout. (**a**) Loading subsystem. (**b**) AE monitoring subsystem. (**c**) AE sensor location. (**d**) Typical example.

The present study focused on the uniaxial compression experiments conducted on fissured sandstones under both natural and water-saturated conditions. As shown in Figure 3, the experimental system consisted of two subsystems: the loading subsystem and the AE monitoring subsystem. The function of the loading subsystem was to perform uniaxial compression on the fissured sandstone. Displacement control was employed as the loading method, with a constant loading rate of 0.002 mm/s [29]. The function of the AE monitoring subsystem was to collect the AE signals generated during uniaxial compression damage of fissured sandstone. The AE system used in this study was the DS5 AE monitoring system developed by Beijing Soft Island Times, China. The frequency range of the AE sensor is 50–400 KHz. To mitigate the signal-to-noise ratio degradation caused by increasing transmission distance during data collection, the original AE signals were amplified by 40 dB using preamplifiers. The amplified signals from all four channels were synchronously collected at a sampling frequency of 2.5 MHz while a collection threshold value of 40 dB was maintained [30]. Subsequently, the acquired signal data underwent postprocessing and were outputted using dedicated analysis software accompanying the equipment. The AE sensor location is shown in Figure 3c. The uniaxial compression test allows the acquisition of stress–strain curves during damage to fissured sandstones. Firstly, we monitored the displacement data of the specimen during compression by LVDT (shown in Figure 3d). Then, based on the specimen size of 50 × 100 mm, we calculated the strain data of the specimen during compression.

### 2.2. Machine Learning Models

The selection of Random Forest, AdaBoost, and Multilayer Perceptron (MLP) algorithms was motivated by their distinct yet complementary capabilities in addressing complex classification tasks. Random Forest is well regarded for its robustness in response to overfitting and its proficiency in managing high-dimensional datasets, offering reliable generalization. AdaBoost is chosen for its iterative approach in refining weak learners, thereby progressively enhancing model accuracy. The MLP, with its deep learning architecture, is adept at capturing intricate nonlinear relationships within the data. These algorithms collectively provide a robust framework for comprehensive model evaluation and performance optimization.

#### 2.2.1. Random Forest

**Figure 4:**
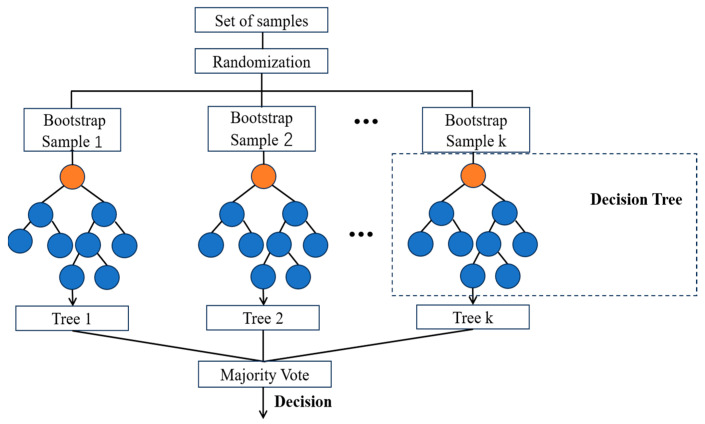
The implementation principle of Random Forest.

Breiman proposed the Random Forest (RF) algorithm for classification and regression [31,32]. Similar to AdaBoost, RF is also an ensemble learning method. In the case of solving classification problems, the Random Forest algorithm determines its output by taking the mode of the outcomes from multiple decision trees. Thus, RF can be considered a classifier consisting of numerous decision trees. The key steps in implementing Random Forest include bootstrap sampling, random feature selection, construction of decision trees, and ensemble method selection [31]. During the process of random sampling, bootstrap sampling is employed to randomly select samples from the training set. As this introduces randomness into the data, each decision tree’s training set becomes unique. Figure 4 shows the implementation principle of the RF.

#### 2.2.2. Multilayer Perceptron

**Figure 5:**
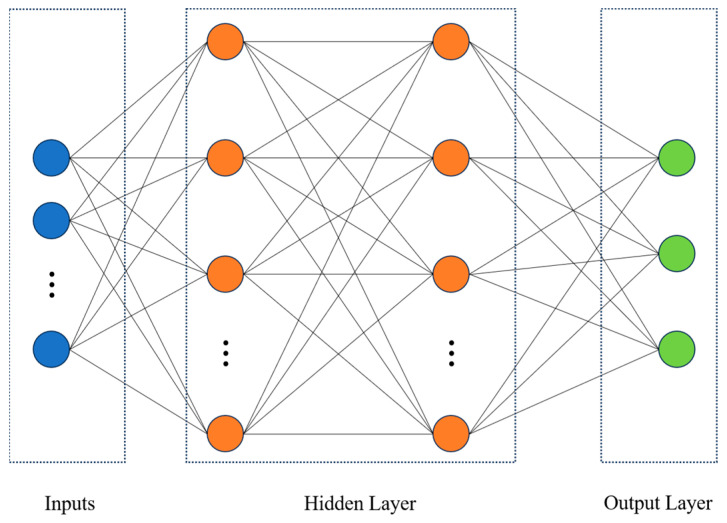
The implementation principle of MLP.

**Figure 6:**
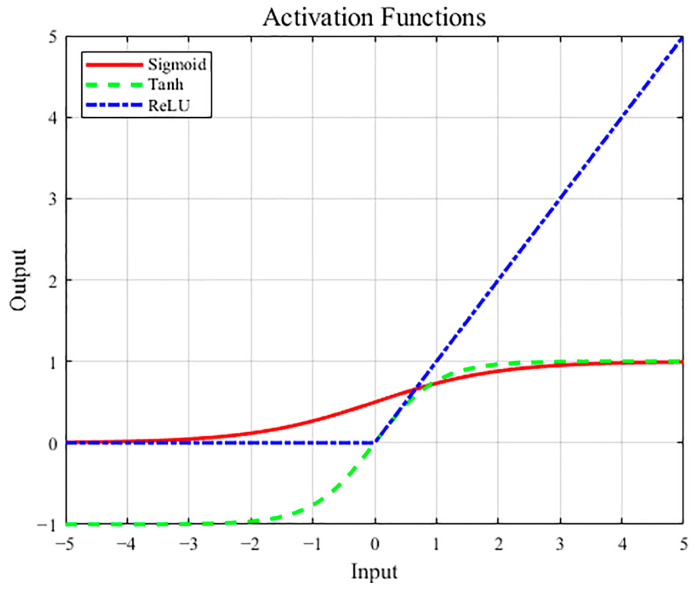
Principles of different activation functions.

The principle of deep learning neural networks aims to mimic the structure and functionality of biological neural networks. It employs mechanisms and methods akin to those found in nature for transmitting and processing feature neural signals, thereby achieving classification outputs for new samples [33]. The Multilayer Perceptron (MLP) is a feedforward neural network model consisting of multiple neurons, which includes an input layer, several hidden layers, and an output layer. In this model, each neuron receives the output from the preceding layer of neurons and undergoes a nonlinear transformation through an activation function before transmitting the result to the subsequent neuronal layer [33,34]. The feedforward propagation of MLP enables it to effectively solve intricate nonlinear problems, as depicted in Figure 5 for the implementation principle of MLP. The hyperparameters associated with hidden layers, including the number of hidden layers, the neural unit count within each hidden layer, and the choice of activation function [33,35], can be adjusted. The prediction outcomes of the MLP model can generally be enhanced by increasing the number of hidden layers and neural units within each layer. However, there exists an upper threshold beyond which further improvement in prediction accuracy is no longer observed. The activation function plays a crucial role in introducing nonlinear operations to both hidden and output layers, thereby enhancing the complexity and expressiveness of the neural network’s output. The selection of an appropriate activation function should be based on data characteristics. Three commonly employed activation functions include sigmoid, tanh, and ReLU [36]. Detailed principles for these three activation functions are illustrated in Figure 6.

#### 2.2.3. AdaBoost

**Figure 7:**
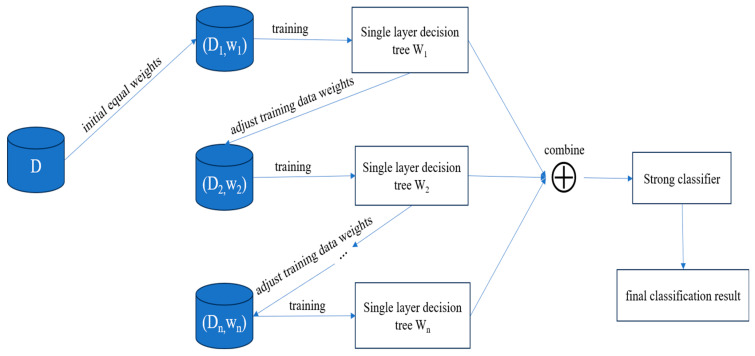
The implementation principle of AdaBoost.

AdaBoost is a typical ensemble learning algorithm proposed by Freund [37,38]. The core idea of ensemble learning is to combine the judgments and opinions of multiple models to minimize the inaccuracy of a single model and produce more stable outcomes across various situations. The primary principle of AdaBoost involves training multiple weak classifiers. This process iteratively updates the weights of subsequent classifiers based on the previous classification results using the same training set. Strong classifiers are synthesized according to the weights assigned to each weak classifier upon the completion of each iteration. Finally, the final weak classifiers are fused into a final strong classifier for binary or multiple classification problems [39]. Figure 7 depicts the implementation principle of AdaBoost.

#### 2.2.4. Model Performance Evaluation Metrics

**Figure 8:**
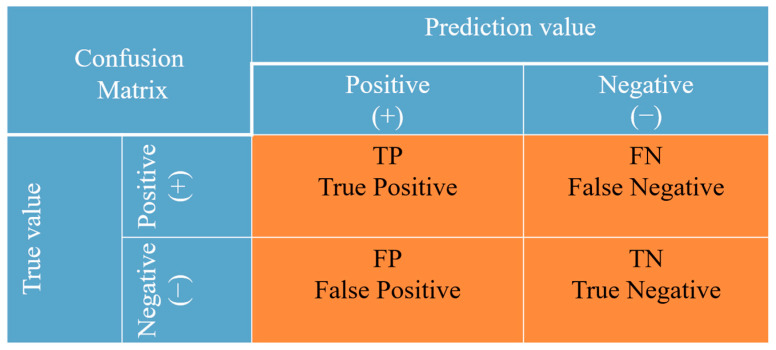
Confusion matrix principle for binary classification problems.

In early warning models for fissured sandstone instability, it is crucial to assess the performance of these models. To validate the performance of the three machine learning models, five validation metrics suitable for classification issues were selected, namely confusion matrix, *accuracy*, *precision*, *recall*, and *F*_1_ score [40,41,42]. The identification results of fissured sandstone risk states were visualized using a confusion matrix. In the context of a standard binary classification problem, the confusion matrix is represented as a 2 × 2 matrix with four possible outcomes [43,44], as shown in Figure 8. The confusion matrix employed in this study for identifying the risk state of fissured sandstone is a 3 × 3 matrix, as shown in Table 1. Each column represents the predicted failure state, while each row corresponds to the true failure state of the data [45].

The most commonly used performance metric for classification is *accuracy* (*acc*), which measures the model’s ability to correctly identify fissured sandstone failure states. In this study, accuracy was calculated by dividing the number of correctly identified samples by the total number of samples. The *precision* (*pre*) represents the percentage of samples classified as positive by the model, indicating its ability to accurately predict true positives. Generally, a higher accuracy and precision indicate an improved model performance. The *recall* measures the ratio of positively classified samples to the total number of positive samples, reflecting how well the model predicts positive instances. The *F_β_* score is a metric used in machine learning to evaluate the performance of a classification model as well. The *F_β_* score offers a weighted average of precision and recall, with the *F*_1_ score treating both metrics as equally important. It is important to note that accuracy and recall are often negatively correlated, indicating a trade-off between the two. To comprehensively evaluate a classifier and strike a balance between accuracy and recall, the *F*_1_ score was introduced as an all-encompassing metric, indicating higher model quality. Accuracy, precision, recall, and *F*_1_ can be employed to assess the overall performance of the model. Additionally, precision, recall, and *F*_1_ can be utilized to measure specific class characteristics as well. These four metrics for evaluating model performance were computed using (1)–(4).

(1) $$acc=\frac{TP+TN}{TP+TN+FP+FN}$$

(2) $$pre=\frac{TP}{TP+FP}$$

(3) $$recall=\frac{TP}{TP+FN}$$

(4) $$F_{1}=\frac{2TP}{2TP+FP+FN}$$

## 3. Analysis of Instability Precursor Information in Fissured Sandstone

### 3.1. Failure Stage Division of Fissured Sandstone

**Figure 9:**
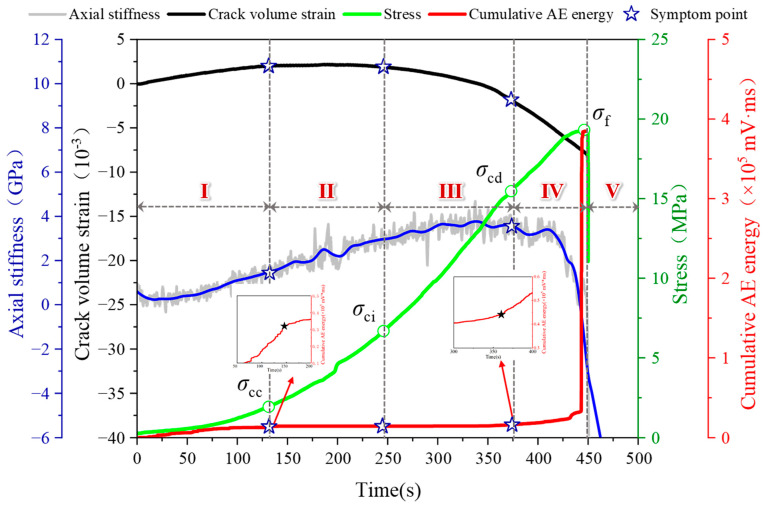
Stage division in the failure process of fissured sandstone.

Since the overall trend of the damage process in the fissured sandstone in the natural and water-saturated states remains the same, we have analyzed only the damage process in the fissured sandstone in the natural state and the AE response during the process. Based on the stress–strain curve and the change in AE cumulative energy, the fracture propagation process in fissured rock under compression can be divided into five stages [29], as depicted in Figure 9.

Stage I: Original microcrack compaction stage. The prominent characteristic of this stage was the concave stress–strain curve. It is evident that the specimen’s closure degree gradually increased with positive compression. The presence of internal particle contacts and frictions led to a significant occurrence of low energy value acoustic emission (AE) activity during this stage. The cumulative AE energy curve exhibited a nonlinear expansion pattern. Subsequently, the stress–strain curve became typically linear, accompanied by reaching its maximum crack volume value. Simultaneously, AE activity entered a quiescent period coinciding with the attainment of crack closure stress σ_cc_.

Stage II: Elastic deformation stage. The stress–strain curve exhibited a linear relationship. During this stage, the applied stress was insufficient to initiate new cracks or propagate existing ones. Consequently, the crack volume strain curve followed a consistent and steady trajectory with minimal AE activities observed. The cumulative AE energy curve remained stable throughout this period. The demarcation point between stage II and stage III, occurring just before the subsequent peak, represents the crack initiation stress, σ_ci_.

Stage III: Stable crack growth stage. The stress level surpassed the threshold at which crack initiation occurred, leading to the emergence of cracks within the specimen. The interior of the specimen underwent a transition from a compression-closed state to a tensioned state, resulting in a decline in the curve depicting volume strain associated with crack propagation. Due to water’s attenuation effect on AE energy propagation speed, there was minimal alteration observed in the cumulative AE energy curve during this period. The applied stress continued to increase, causing continuous expansion of cracks within the specimen and gradual escalation of damage severity. Consequently, both the stress–strain curve and axial stiffness curve exhibited deviations from linearity. The demarcation point between stages III and IV was defined as σ_cd_—critical stress for crack damage.

Stage IV: Accelerated crack growth stage. A concave trend was observed in the stress–strain curve, indicating the initiation of yielding and deformation in the specimen. The expansion and merging of internal microcracks formed a network, resulting in the occurrence of the stress drop phenomenon. Concurrently, high-energy AE events were detected, leading to a steep increase in the cumulative AE energy curve. Subsequently, the peak strength σ_f_, representing the maximum applied stress level, was attained.

Stage V: Macro damage stage. In stage V, the stress exhibited a peak strength after the cliff fall, with the descent reaching 50% of the maximum stress level. Notably, discernible macroscopic damage was observed in the specimen.

### 3.2. AE Energy and AE Ringing Count Characterization

**Figure 10:**
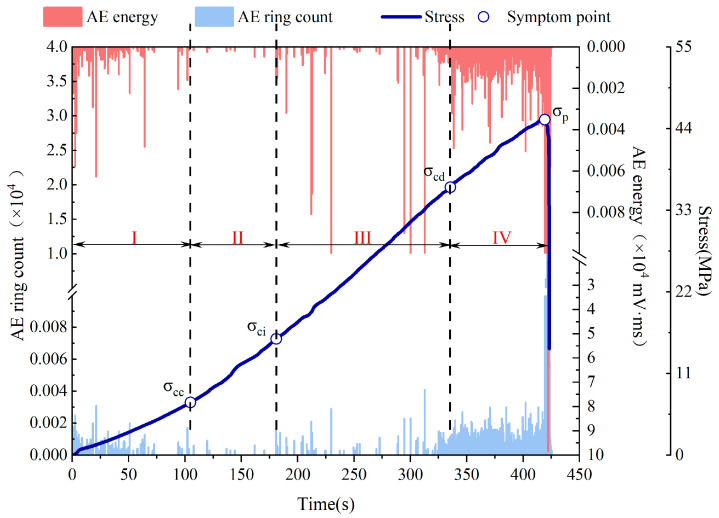
Evolution law of AE energy and AE counts in the failure process of fissured sandstone.

The degree of rock mass damage can be inferred from the energy value of AE. Microfractures are associated with low-energy AE events, while macroscopic cracks are associated with high-energy AE events. Therefore, the magnitude of the AE energy value could indicate the strength of rock body rupture. Additionally, it characterizes intrinsic structural changes in the deformation and damage of fissured rock bodies. Figure 10 illustrates the evolution law of AE energy value and AE ringing count during fissured sandstone damage. Based on Figure 10, the evolution patterns of AE energy and AE ringing count showed a strong consistency between the natural and saturated states in general. It can be inferred that during stage I, there was a frequent occurrence of AE events with low energy values and ringing counts, which were closely associated with the compression and closure of internal original microcracks. In stage II, a noticeable decline in both AE energy value and ringing count was observed, indicating a period of relative quiescence for AE events. During stage III, the AE events became active again as stable crack expansion commenced in the specimen. Finally, in stage IV, there was an intensive occurrence of AE events characterized by high energy values and ringing counts.

### 3.3. Centroid Frequency and Peak Frequency Characterization

**Figure 11:**
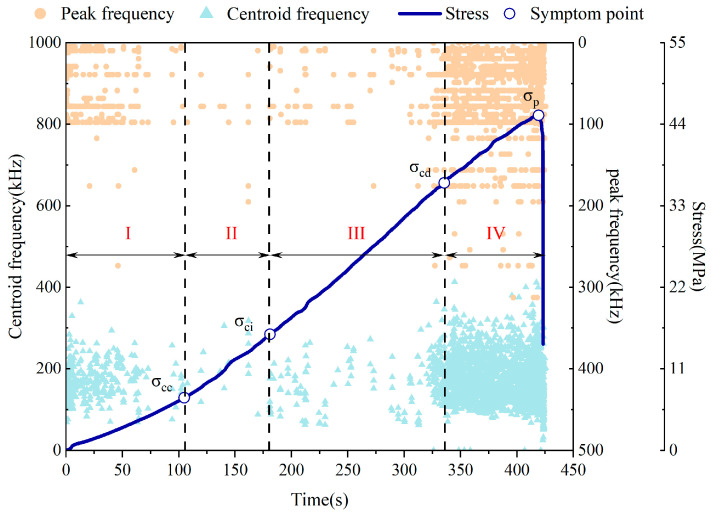
Evolution law of centroid frequency and peak frequency in the failure process of fissured sandstone.

Both centroid frequency and peak frequency are crucial characteristic parameters for characterizing the nature of the rupture source [10]. The former quantifies the proportion of high-frequency energy, while the latter is commonly employed to typify the type of rupture source. These two parameters can be effectively utilized to discern alterations in the internal conditions of fissured sandstone. The results presented in Figure 11 demonstrate that during the initial loading phase, the internal fracture form of fissured sandstone was predominantly characterized by original microcrack compaction. During this stage, small-scale and low-frequency AE signals were found to be dominant, while medium–high-frequency signals were rarely observed. As the crack growth occurred within the fissured sandstone, the AE signals reverted to exhibiting characteristics of small-scale and low-frequency events. Subsequently, upon entering stage III, there was a noticeable intensification in both medium- and high-frequency signals. Furthermore, with continued loading, there was a significant increase in the proportion of medium- and high-frequency signals detected. Consequently, it can be inferred that the emergence of medium- and high-frequency signals serves as precursor information for accelerated crack propagation within fissured sandstone.

### 3.4. RA Value and AF Value Characterization

**Figure 12:**
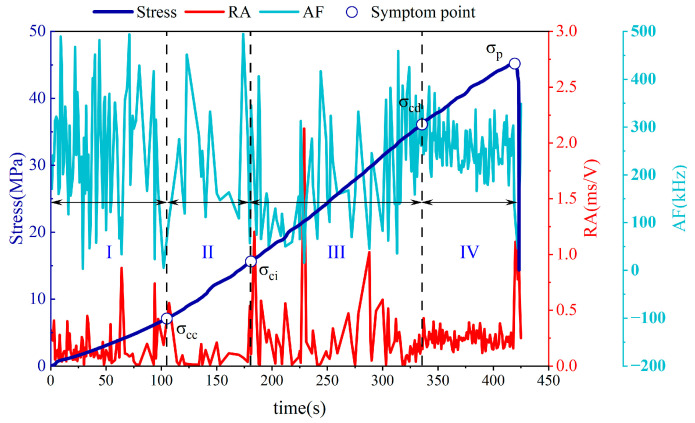
Evolution law of RA and AF in the failure process of fissured sandstone.

The Rise Angle (RA) value is the ratio of rise time (RT) to amplitude (A). The Average Frequency (AF) value stands for Average Frequency, which is the ratio of the event frequency of the acoustic emission signal to the ring-down count (RC). The combined use of RA and AF values provides a more comprehensive analysis of crack propagation behavior in materials or structures. The evolution pattern of RA and AF values with time during the fissured sandstone damage process is presented in Figure 12. It can be observed from Figure 12 that both the RA value and AF value exhibited an overall fluctuating trend over time. Initially, during the loading phase, both values underwent fluctuations at a certain level. Subsequently, as stable crack growth occurred before reaching the peak, it is noted that the AF value started to increase while the RA value remained low and changed smoothly. Upon reaching the crack damage stress point, there was a significant flattening of fluctuations in both the RA value and the AF value, accompanied by a decrease in AF value fluctuation and an increase in RA value fluctuation. This phenomenon can be considered as characteristic of stage III—“fluctuating increase of AF value and low-steady change of RA value”. The inclination towards decreased fluctuation in AF value along with an increased rise in RA value can serve as early indicators for accelerated crack growth.

### 3.5. b Value Characterization

The AE *b* value is an indicator of the microfracture scale within a brittle rock body. It can reflect the evolution of microcracks during the damage process [46]. A decrease in the *b* value typically indicates a reduction in low-amplitude AE events associated with microfractures. Conversely, an increase in high-amplitude AE events suggests significant rupture. When the *b* value fluctuates within a certain range, it signifies stable rock body rupture occurrence. However, a sharp decline in the *b* value over a short period implies a sudden increase in large fracture events and accelerated crack propagation, potentially leading to instability failure [47]. Originally proposed based on the earthquake frequency–magnitude relationship in seismology, the AE *b* value calculation replaces seismic magnitude with AE amplitude after reducing it by 20 times to match magnitude values. Equation (5) presents the calculation formula for determining the AE *b* value.

(5) lgN=a−b(A/20)

**Figure 13:**
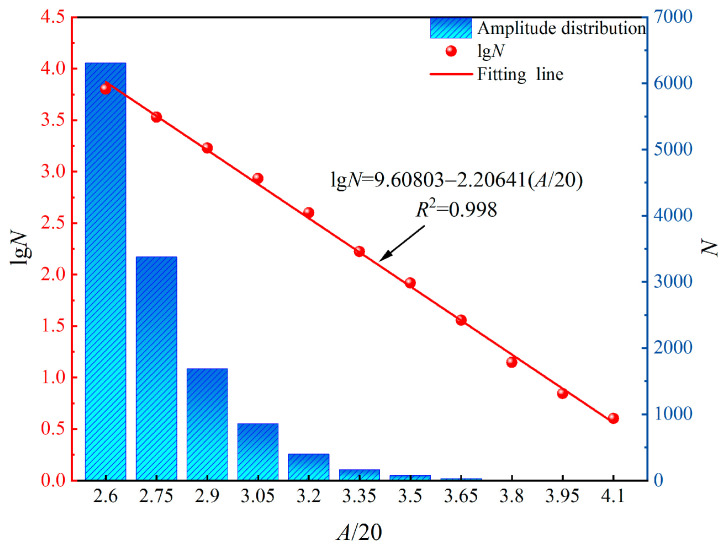
AE amplitude distribution of fissured sandstone.

The AE event amplitude, denoted as *A* in this study, is measured in decibels (dB). *N* represents the cumulative number of AE events with an amplitude greater than *A*. The amplitude interval used for analysis was set at 3 dB. Refer to Figure 13 for a detailed illustration of the specific statistical process. The least squares method was employed to calculate the *b* value, based on which a statistical window of 2000 AE events and a sliding step size of 500 events were used for sampling calculation [47].

**Figure 14:**
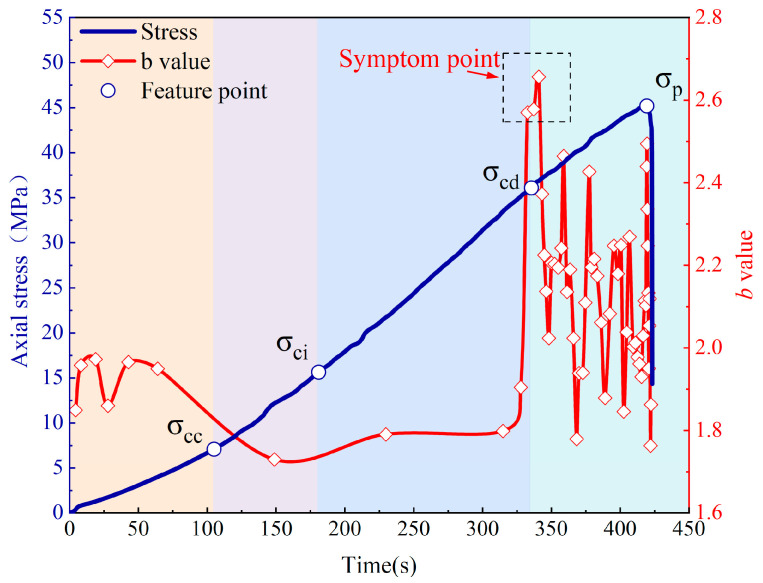
Evolution law of *b* value in the failure process of fissured sandstone.

The variation in the *b* value with time during the loading process of fissured sandstone is shown in Figure 14. In stage I, the *b* value fluctuated within a narrow range as the interior structure of the fissured sandstone adjusted. During stage II, there was a slight increase in the *b* value followed by stabilization, indicating an increase in microfracture events and the initiation of cracking in the fissured sandstone. As the damage stress approached its threshold, there was a sharp increase in the *b* value accompanied by intensive microfracture events within the fissured sandstone. These microfracture events accumulate continuously to generate larger fracture events. Upon reaching the damage stress level, there was a rapid decrease in the *b* value and accelerated growth of cracks, leading to violent changes in the internal structure of the fissured sandstone. Subsequently, fluctuations and changes were observed in the *b* value. At the same time, cracks at different scales penetrated to form macroscopic fracture surfaces. Therefore, it can be considered that a sharp decline in the *b* value serves as precursor information for instability occurrence in fissured sandstone.

## 4. Development of Instability State Identification Model Based on Precursor Information

### 4.1. Data Acquisition

A uniaxial compression was utilized apparatus to perform uniaxial compression tests on fissured sandstones. Two fissured sandstones (10 mm length) under natural and water-saturated conditions, respectively, were adopted as objects of study. The AE signals of the whole process of uniaxial compression damage in fissured sandstone were collected using the AE monitoring system. The complete AE signals were used for further data mining.

### 4.2. Data Preprocessing

In machine learning, data preprocessing is a pivotal step that precedes the model training phase. It involves a series of techniques aimed at transforming raw data into a format that is more suitable for the algorithms to learn from. Specifically, in this study, to avoid the influence of noisy data on the results during data acquisition, the parameter values were averaged over 1 s time intervals. At the same time, data normalization was used to eliminate the effects of different magnitudes on the model [41].

### 4.3. Dataset Establishment

The label used for machine learning models was the instability risk of the fissured sandstone at that specific 1 s moment. We categorized stage I and stage II as Label 0 (indicating no risk of instability), stage III as Label 1 (indicating a medium risk of instability), and stage IV and stage V as Label 2 (indicating high risk of instability). After data preprocessing, the data was combined with labels to form the final dataset. Our dataset consisted of 585 data points with 291 collected under natural conditions and 294 under saturated conditions. In this dataset, 261 pieces of data were labeled as 0, 154 pieces of data were labeled as 1, and 170 pieces of data were labeled as 2. The dataset was well balanced.

### 4.4. Dataset Distribution

Distribution histograms serve as a visual tool for illustrating the distribution of numerical data, playing a vital role in data analysis by depicting the overall form and dispersion of a dataset. Hence, histograms were created to visualize the above dataset. This can help us better understand the internal distribution of data. While observing the distribution of the data, it is possible to understand the statistical differences in the features and obtain a side-by-side explanation of the feasibility of a feature being used as a machine learning input feature. Two perspectives were chosen to analyze the distribution of each parameter in the dataset. One was the distribution of each parameter in the natural and saturated states. The other was the distribution of each parameter in three different risk states for damage destruction of fissured sandstone.

#### 4.4.1. Data Distribution of Different States

**Figure 15:**
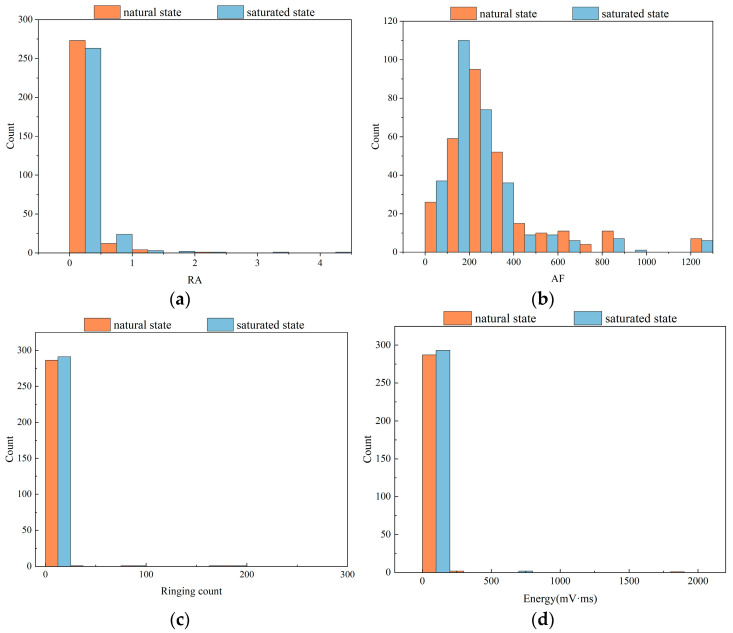

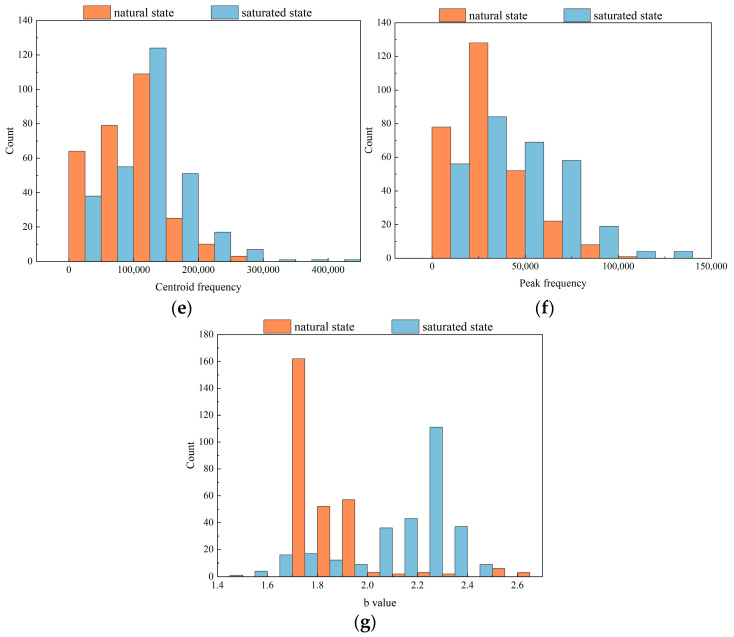
Distribution of AE parameters for damage processes in fissured sandstones under natural and saturated states. (**a**) RA. (**b**) AF. (**c**) Ringing count. (**d**) Energy (mV·ms). (**e**) Centroid frequency. (**f**) Peak frequency. (**g**) *b* value.

Figure 15 shows the distribution of each feature in the natural state and saturated state. The following information can be obtained: Each parameter showed a consistent distribution pattern in both natural and saturated states. However, except for the ringing count and energy, the rest of the features showed significant differences between the natural and saturated states under the same distribution pattern. Taking the *b* value as an example, the *b* value of the natural fissured sandstone was mainly concentrated in the range of 1.7 to 2.0, whereas under the saturated condition, it was concentrated in the range of 2.0 to 2.4 and more uniformly distributed than that of the natural condition. The significant differences indicated that these parameters were suitable to be used as input parameters for machine learning models.

#### 4.4.2. Data Distribution of Different Instability Risk States

**Figure 16:**
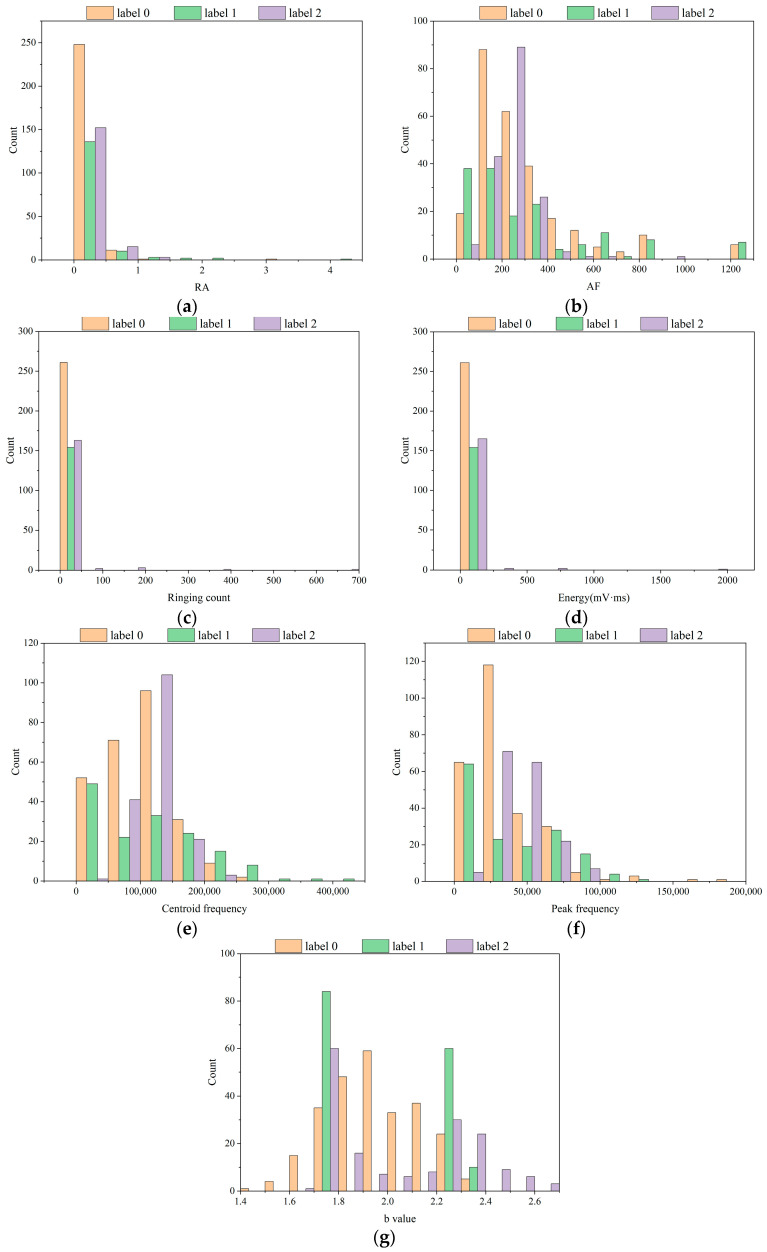
Distribution of AE parameters in different instability risk states for fissured sandstone. (**a**) RA. (**b**) AF. (**c**) Ringing count. (**d**) Energy (mV·ms). (**e**) Centroid frequency. (**f**) Peak frequency. (**g**) *b* value.

Similar to the analysis above, as shown in Figure 16, the overall statistical trends of individual precursor information were consistent in the natural and saturated states. Label 0, Label 1, and Label 2 represent no instability risk state, medium instability risk state, and high instability risk state, respectively. We take the *b* value as an example: The *b* value of the no instability risk state ranged from 1.4 to 2.4, and the center of gravity was around 1.9. The *b* value in the medium instability risk state was mainly concentrated at 1.8 and 2.3, with a small distribution in the range of 1.8 to 2.3. The *b* value for the high instability risk state was distributed between 1.8 and 2.7, and the data were more dispersed than those in the remaining two states. Once again, the selected precursor information indicators were justified.

### 4.5. Dataset Splitting

**Figure 17:**
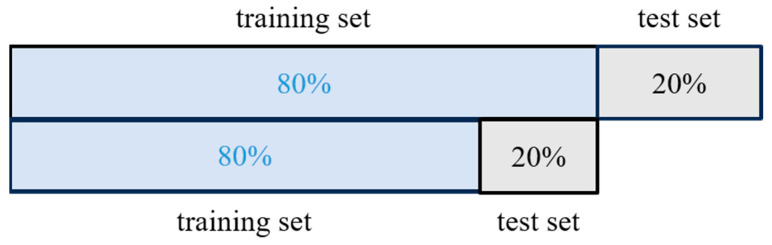
Dataset splitting method.

Figure 17 illustrates the method of segmenting the dataset into a training set comprising 80% of the data and a test set comprising the remaining 20%. Within the training set, 80% were used for model training while the remaining portion served as validation data [35,36].

### 4.6. Hyperparameter Splitting

The default parameters do not typically yield optimal performance for the model. Poorly performing models often suffer from a significant generalization error when faced with unidentified data. This error stems from an excessive complexity in the model, leading to substantial generalization mistakes. Striking a balance between overfitting and underfitting is crucial for achieving high accuracy in the model. Therefore, it is imperative to adjust the model’s parameters based on circumstances to enhance its accuracy significantly. Table 2, Table 3 and Table 4 present specific parameters for the three ML models established in this paper.

### 4.7. Model Training

**Figure 18:**
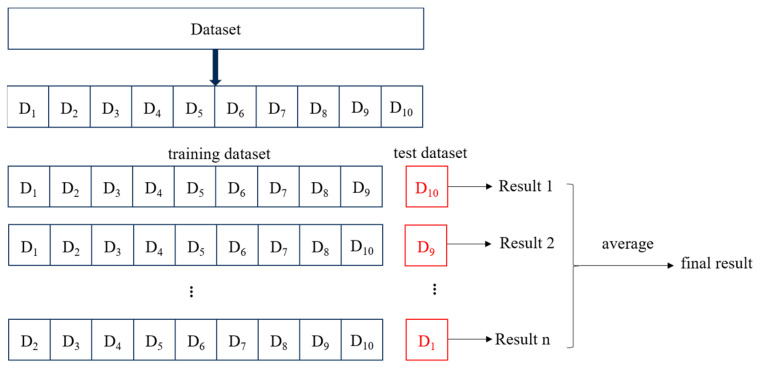
Schematic diagram of ten-fold cross-validation.

By employing randomly generated subsamples for training and validation in a repeated manner using the 10-fold cross-validation method depicted in Figure 18, model accuracy and enhanced dataset utilization were significantly improved. After the above steps, machine learning models could be trained using the built dataset. The trained models were highly discriminative for unknown data, reducing a lot of redundant and repetitive work.

## 5. Performance Analysis and Input Feature Valuation of Machine Learning Models

### 5.1. Models’ Performance

**Figure 19:**
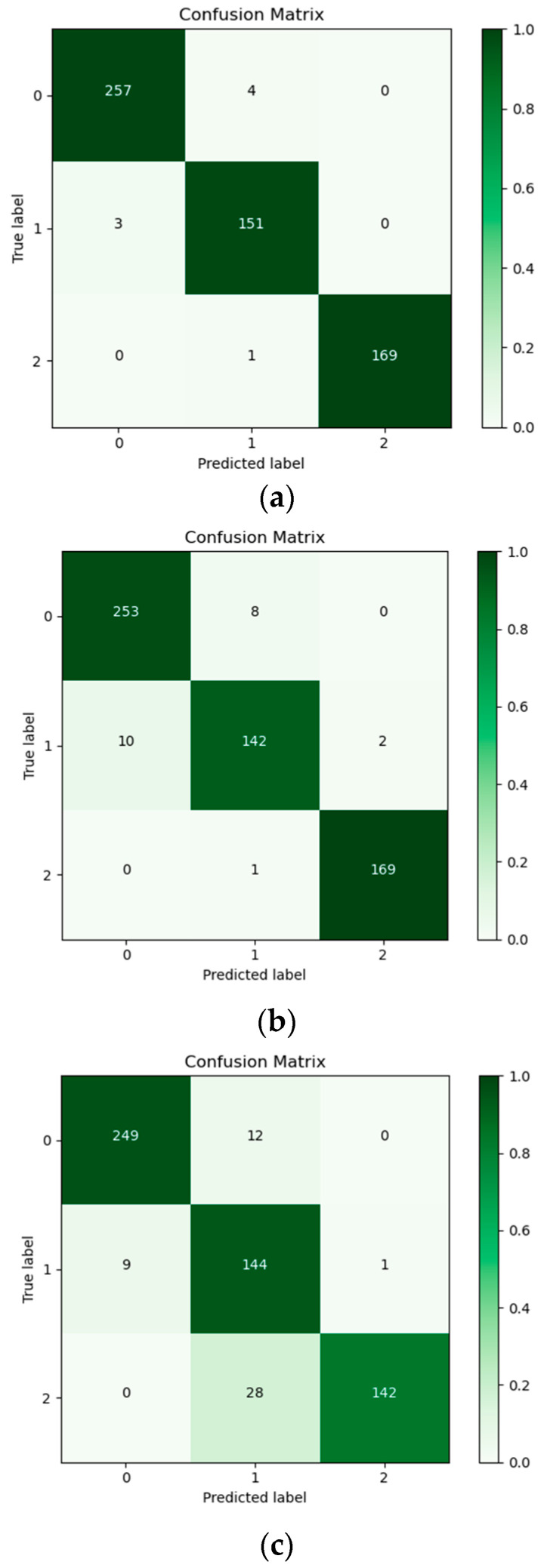
The confusion matrices of three distinct models for identifying instability in fissured sandstone. (**a**) Random Forest. (**b**) MLP. (**c**) AdaBoost.

The performance metrics of the three fissured sandstone instability risk identification machine learning models we established were analyzed in detail. Figure 19 shows the visualized confusion matrices for the three models. From the figure, we can see that the higher the accuracy, the closer the color was to dark green, and the lower the accuracy the closer the color was to light green or even white. The number in the box indicated, the number of data in that condition. Based on the confusion matrix heat map, a preliminary conclusion can be drawn. The Random Forest model had the best performance in solving the problem of damage state identification of fissured sandstone, followed by the MLP model, and the AdaBoost model had the worst classification performance.

**Figure 20:**
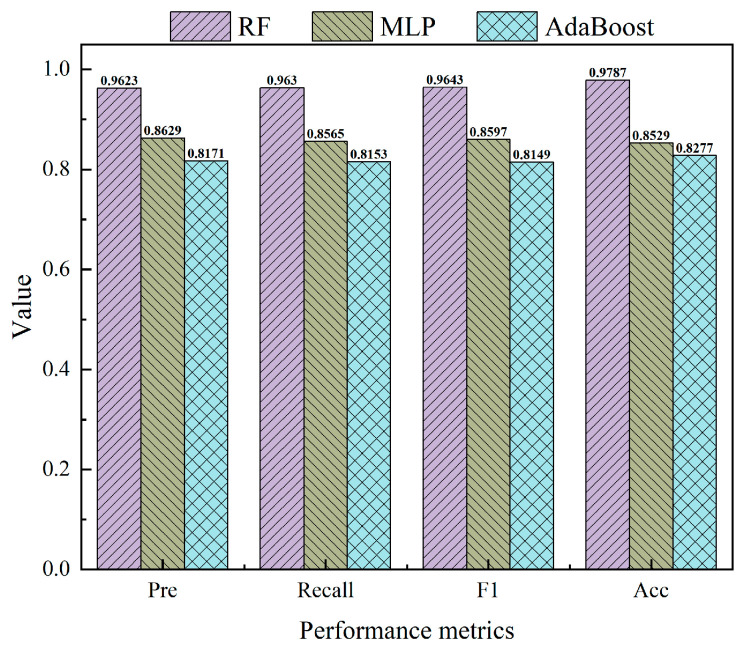
Overall performance metrics of the fissured sandstone instability risk identification models.

**Figure 21:**
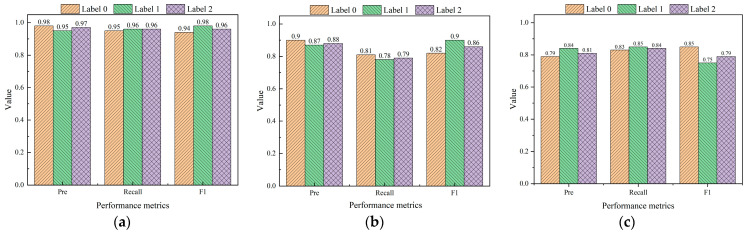
Performance metrics of the fissured sandstone instability risk identification models under different instability risks. (**a**) Random Forest. (**b**) MLP. (**c**) AdaBoost.

In conjunction with Figure 20, the following information was available: Among the three ML classification methods, the RF algorithm exhibited superior performance in terms of *accuracy*, *precision*, *recall*, and *F*_1_ values, achieving scores of 0.9787, 0.9623, 0.9630, and 0.9643, respectively. It was subsequently followed by MLP, while AdaBoost demonstrated comparatively weaker performance. By evaluating the performance of these algorithms across three categorization outcomes (as shown in Figure 21), it can be concluded that the RF algorithm consistently outperformed its counterparts. It is noteworthy that the RF model had optimal performance, especially in the identification of medium and high instability risks. This is vital for application to realistic working conditions. The findings indicated that for the dataset that we employed, the RF algorithm was deemed most suitable. That is, the risk of instability of fissured sandstone predicted by the RF model was the most reliable.

### 5.2. Importance Analysis of Model Input Features

Feature importance analysis is an approach commonly employed in machine learning models to assess the significance or value of each input parameter for predicting outcomes [48,49]. This analysis enables the identification of the key factors that exert the most influence on model outputs. Moreover, feature importance analysis can enhance model performance, mitigate overfitting issues, and improve interpretability. Among the three machine learning models established in this study, the RF model demonstrated superior performance. Therefore, the importance of the parameters in the RF model was analyzed. To ascertain the significance of different parameters’ impact on results within the RF model, the ‘feature_importance’ function [36] in machine learning was utilized.

**Figure 22:**
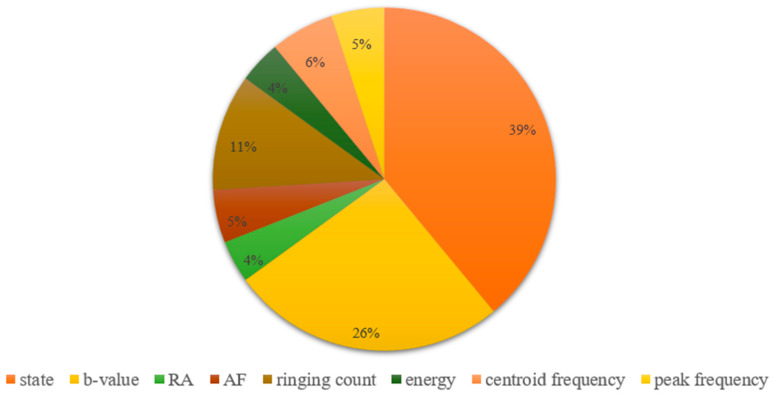
The importance of each input feature in the Random Forest model.

The significance of the eight selected parameters for identifying instability risk states in fissured sandstone is demonstrated in Figure 22. Among these parameters, state and *b* value exhibited the highest contribution to the identification results, followed by AF and RA. Ringing count, energy, peak frequency, and centroid frequency contributed to a similar extent at approximately 5%. This result was consistent with the analysis of the data distribution and again justified the selection of features in this study. Notably, the combined influence of state and *b* value exceeded 60% on the classification outcomes. Therefore, state and *b* value were considered primary criteria in the model for identifying instability risk in fissured sandstone.

The analysis of feature importance holds significant value in determining the contribution rate of individual parameters to classification results. However, it also possesses certain limitations as it fails to capture the interrelationships between parameters. To address this issue and examine parameter correlations, the Spearman correlation coefficient was introduced.

### 5.3. Correlation Analysis of Model Input Features

Spearman’s rank correlation coefficient is a non-parametric statistical measure that assesses the monotonic relationship between two variables. It evaluates the degree of positive or negative correlation by comparing the rank orders of the variables rather than their actual values. The Spearman rank correlation coefficient is defined as follows [50]:

(6) $$\rho =1-\frac{6\sum_{i}^{n}{d_{i}}^{2}}{n(n^{2}-1)}$$

**Figure 23:**
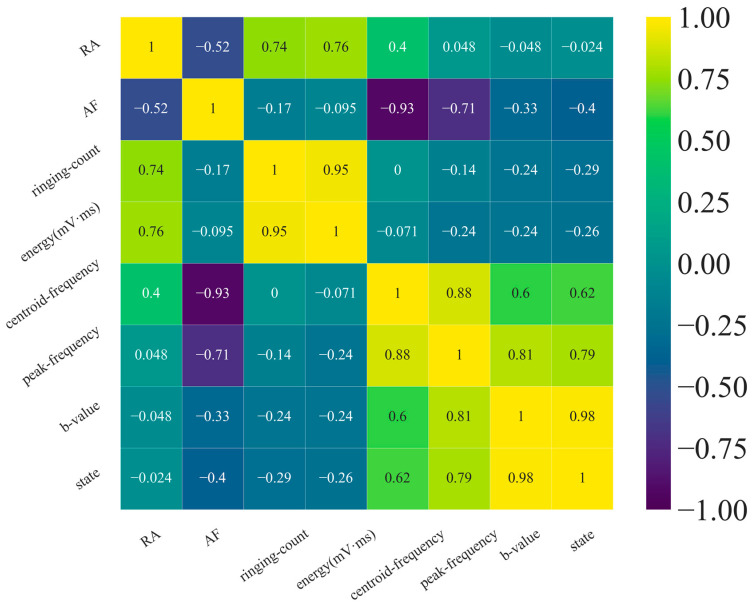
Spearman correlation coefficient between input features.

The Spearman correlation coefficient was computed for the eight parameters utilized as inputs in the machine learning models. A heat map, depicted in Figure 23, was generated to visualize these correlations. The Spearman correlation coefficient ranged from −1 to 1, with values closer to 0 indicating weaker associations between the parameters. Positive correlations were observed when the coefficient exceeded 0, while negative correlations were observed when it fell below 0. Correlations with coefficients less than 0.05 were considered statistically insignificant. As the parameters were pairwise calculated for their Spearman correlation coefficients and exhibited positive self-correlation, the resulting heat map represents a symmetric matrix with a diagonal of unity. For example, the final column of the matrix displayed the correlation between the state and the remaining parameters. It was observed that state was nearly no correlation with RA value, a weak negative correlation with ringing count and energy (mV·ms), a moderate negative correlation with AF, and a strong positive correlation with centroid frequency, peak frequency, and *b* value.

In machine learning, the smaller the correlation between parameters, the better the model performance. The results of the correlation analysis showed that the correlation between most of the features we selected was relatively small. It indicated that the overall feature selection in this study was reasonable. However, there were also some parameters with high correlation between them. The subsequent research can adopt more effective feature engineering methods to reduce the correlation between the features with high correlation based on the results of this study, further improving the performance of the machine learning model.

## 6. Conclusions

The relationship between AE parameters and damage characteristics was established in this study, elucidating the multi-parameter information associated with instability precursors of fissured sandstone under natural conditions and water–rock interaction. Additionally, machine learning algorithms were employed to construct models for identifying the instability state of fissured sandstone under both natural conditions and water–rock interaction. The key findings are as follows:

(1) Based on the crack extension process, the fissured sandstone damage was divided into five stages. The stage-wise evolutionary patterns of AE parameters, including ringing count, energy value, centroid frequency, peak frequency, RA value, AF value, and *b* value, during the uniaxial compression process of fissured sandstone were analyzed.

(2) A dataset that can be used by machine learning models was created. Three machine learning models for identifying the risk of instability in fissured sandstones were developed. The optimal parameters for each machine learning model were found by grid search. It was found that the Random Forest model performs best after reaching the optimal performance.

(3) The performances of the three machine learning models built were evaluated. The results showed that the RF model outperformed the remaining two models overall and for different risk states. It indicated that the prediction results of the models built based on the RF algorithm had the highest confidence for the problem of identifying the instability state of fissured sandstone.

(4) The value of each input feature of the RF model was analyzed in terms of both parameter importance and correlation. The results of parameter importance analysis showed that the natural/saturated state of the fissured sandstone and the AE *b* value contributed the most to the results and exceeded 60%. The results of parameter correlation analysis showed that most of the features used had less correlation with each other, which justified the feature selection.

In the future, more AE statistical parameters can be mined through further feature engineering as precursor information for the instability of fissured sandstone under water–rock interaction. These new parameters can be used as input features for machine learning to further improve the accuracy of the instability risk identification of fissured sandstone. Furthermore, more advanced fusion algorithms can be used to improve the speed of machine learning model training and prediction performance [26,27].

## Figures and Tables

**Table 1:** Confusion matrix for fissured sandstone instability identification model.

Confusion Matrix		Predicted Label		
		0	1	2
True label	0	00	01	01
	1	10	11	12
	2	20	21	22

**Table 2:** Parameter settings in the Random Forest model.

Parameter	Parameter Set for This Model
n_estimators	5
max_depth	10
min_samples_leaf	1
min_samples_split	2
class_weight	balanced
criterion	gini

**Table 3:** Parameter settings in the MLP model.

Parameter	Parameter Set for This Model
hidden_layer_sizes	(30,20)
activation	adam
solver	adam
alpha	0.1
max_iter	400

**Table 4:** Parameter settings in the AdaBoost model.

Parameter	Parameter Set for This Model
base_estimator	CART decision tree
n_estimators	300
learning_rate	0.6
algorithm	SAMME
random_state	37

## Data Availability

The experimental data used to support the findings of this study are included within the article.
